# Scores TISS-28 *versus* NEMS to size the nursing team in a pediatric intensive care unit

**DOI:** 10.1590/S1679-45082017AO4028

**Published:** 2017

**Authors:** Kelly Dayane Stochero Velozo, Pedro Celiny Ramos Garcia, Jefferson Pedro Piva, Humberto Holmer Fiori, Daiane Drescher Cabral, Paulo Roberto Einloft, Francisco Bruno, Cristian Tedesco Tonial, Caroline Abud Drumond Costa, Simone Travi Canabarro

**Affiliations:** 1 Pontifícia Universidade Católica do Rio Grande do Sul, Porto Alegre, RS, Brazil; 2 Universidade Federal do Rio Grande do Sul, Porto Alegre, RS, Brazil; 3 Universidade Federal de Ciências da Saúde de Porto Alegre, Porto Alegre, RS, Brazil

**Keywords:** Nursing staff, Personnel management, Pediatric nursing, Indicators, Workload, Intensive care units, Recursos humanos de enfermagem, Administração de recursos humanos, Enfermagem pediátrica, Indicadores, Carga de trabalho, Unidades de terapia intensiva

## Abstract

**Objective:**

To estimate the workload and size the nursing team using the scales TISS-28 and NEMS in a pediatric intensive care unit.

**Methods:**

An observational prospective study with a quantitative approach was conducted at the pediatric intensive care unit of a university hospital from Jan 1st, 2009 to Dec 31st, 2009. All children who remained hospitalized for more than 8 hours were included, with length of stay of 4 hours in case of death. Clinical data were collected and the Paediatric Index of Mortality 2 and the scores TISS-28 and NEMS were determined. The TISS-28 and NEMS were converted into working hours of the nursing team and sizing complied with the parameters of the Brazilian Federal Nursing Council. Pearson's correlation and the Bland-Altman model were used to verify the association and agreement between the instruments.

**Results:**

A total of 459 children were included, totaling 3,409 observations. The average values for the TISS-28 and NEMS were 20.8±8 and 25.2±8.7 points, respectively. The nursing workload was 11 hours by TISS-28 and 13.3 hours by NEMS. The estimated number of professionals by TISS-28 and NEMS was 29.6 and 35.8 professionals, respectively. The TISS-28 and NEMS showed adequate correlation and agreement.

**Conclusion:**

Time spent in nursing activities and team sizing reflected by the NEMS were significantly greater when compared to the TISS-28.

## RESUMO

**Objetivo:**

Estimar a carga de trabalho e dimensionar a equipe de enfermagem utilizando as escalas TISS-28 e NEMS em uma unidade de terapia intensiva pediátrica.

**Métodos:**

Estudo prospectivo observacional com abordagem quantitativa, realizado na unidade de terapia intensiva pediátrica de um hospital universitário, no período de 1o de janeiro de 2009 a 31 de dezembro de 2009. Foram incluídas todas as crianças que permaneceram internadas por mais de 8 horas, com duração de internação de 4 horas em caso de óbito. Foram coletados os dados clínicos, e determinados o *Paediatric Index of Mortality* 2 e as escalas TISS-28 e NEMS. O TISS-28 e o NEMS foram convertidos em horas de trabalho da equipe de enfermagem, e o dimensionamento seguiu os parâmetros do Conselho Federal de Enfermagem. A correlação de Pearson e o modelo de Bland-Altman foram utilizados para verificar a associação e a concordância entre os instrumentos.

**Resultados:**

Foram incluídas 459 crianças, totalizando 3.409 observações. As médias do TISS-28 e do NEMS foram 20,8±8 e 25,2±8,7 pontos, respectivamente. A carga de trabalho de enfermagem foi de 11 horas pelo TISS-28 e 13,3 horas pelo NEMS. A estimativa do número de profissionais pelo TISS-28 e NEMS foi de 29,6 e 35,8 profissionais, respectivamente. O TISS-28 e o NEMS apresentaram correlação e concordância adequadas.

**Conclusão:**

O tempo despendido nas atividades de enfermagem e o dimensionamento da equipe refletido pelo NEMS foram significativamente maiores quando comparados ao TISS-28.

## INTRODUCTION

Pediatric intensive care units (ICU) provide sophisticated care for critically ill children based on complex therapeutic and technological resources. In this demanding scenario, adequate nurse staffing is essential to ensure the effectiveness of care.^(^ ^1^ ^,^ ^2^ ^)^

In the literature, the complexity of care has been associated with the need for a higher number of nursing professionals per patient.^(^ ^3^ ^)^ Determining the number of staff and the workload required to provide quality care in the pediatric ICU is a challenging task, which is currently addressed in many institutions by the use of scales.^(^ ^4^ ^)^

Two scales originally developed for adult populations,^(^ ^4^ ^–^ ^6^ ^)^ the Therapeutic Intervention Scoring System-28 (TISS-28) and the Nine Equivalents of Nursing Manpower Use Score (NEMS), are often employed to determine patient severity (which would indicate the need for more staff) and to estimate the workload of nursing staff in ICU.^(^ ^7^ ^–^ ^9^ ^)^ To this end, the TISS-28 and the NEMS try to quantify the amount of time spent by each nursing professional on direct patient care activities ( *e.g* ., excluding administrative tasks or counseling) that are typical of the ICU over 24 hours. Such a measure would allow the ICU to calculate the number of nursing professionals required for each shift, and the number of hours each professional should work without compromising patient safety.

The TISS-28 is the older of these two scores. It includes 28 items that cover seven major categories: basic activities, ventilatory support, cardiovascular support, renal support, neurological support, metabolic support, and specific interventions.^(^ ^7^ ^)^ The NEMS, which is simpler and more objective,^(^ ^4^ ^,^ ^8^ ^,^ ^10^ ^,^ ^11^ includes nine items from the TISS-28: basic monitoring, intravenous medication, mechanical ventilatory support, supplementary ventilatory care, single vasoactive medication, multiple vasoactive medication, dialysis techniques, specific interventions in the ICU and specific intervention outside the ICU.^(^ ^8^ ^)^ In practical terms, to produce reliable measurements, nursing personnel using these scales must be knowledgeable about how to interpret each item. Thus, simplicity becomes an important feature, which might increase the applicability and reliability of results.

## OBJECTIVE

To compare the ability of TISS-28 and NEMS indicators to estimate the workload and size the nursing team, and to assess the correlation and agreement between these scores in a pediatric intensive care unit setting.

## METHODS

This observational, longitudinal, prospective, concurrent and comparative study was conducted at the *Hospital São Lucas* , of *Pontifícia Universidade Católica do Rio Grande do Sul* , Porto Alegre (RS), Brazil. The pediatric ICU had 12 active beds and a monthly average of 34 patients, age ranging from 28 days to 18 years of age, who had acute or chronic conditions or were surgery patients. This unit admitted patients from referring hospitals and through the emergency room (external referrals), in addition to those from several specialist wards within the hospital (internal referrals). Access to care was provided through private health insurance or the Brazilian Unified Health System (SUS - *Sistema Único de Saúde* ), which is a government-funded universal health care system that includes the public provision of core physician and hospital services without copayments or patient charges.

The study population sample included all children and adolescents who remained hospitalized for more than 8 hours, with length of stay of 4 hours in case of death, admitted to the pediatric ICU between January 1st, 2009, and December 31st, 2009. Patients readmitted after discharge were counted as new admissions.

During the study period, the pediatric ICU was staffed by 46 nursing professionals divided into four teams: 12 in the morning (6 hours), 12 in the afternoon (6 hours), and two teams of 11 working in 12-hour night shifts (night shift 1 and night shift 2). Considering sick leaves, vacation and time off, nine workers were usually available for each shift. The team for each shift included two registered nurses, as well as auxiliaries and nursing technicians.

TISS-28,^(^ ^7^ ^,^ ^12^ ^)^ demographic, and clinical data were collected prospectively by unit nurses. The data were daily collected by four registered nurses throughout the entire hospitalization of each child, between 12:00pm and 2:00pm, consisting of the patients' medical records from the last 24 hours of hospitalization. All nurses involved in data collection were familiarized with and trained in the use of the instrument, which had been previously used in the pediatric ICU.

TISS-28 items were collected with respect to routine treatment over 24 hours of hospitalization for each patient. The NEMS items were extracted from the TISS-28,^(^ ^8^ ^,^ ^11^ ^)^ Each day was considered a single observation. Disease severity was estimated using the risk score Pediatric Index of Mortality (PIM) 2,^(^ ^13^ ^)^ which was administered by the unit physicians.

The collected data were stored in Microsoft Excel and analyzed with *Statistical Package for the Social Science* (SPSS), version 17.0. The χ^2^ test was used to determine associations between categorical variables, and the Student's *t* test was used for continuous variables; p<0.05 was considered statistically significant. The association and agreement between NEMS and TISS-28 results were calculated using the Pearson correlation and the Bland-Altman model, respectively.^(^ ^14^ ^)^ Flora z test^(^ ^15^ ^)^ was used to compare the general similarity between observed and expected mortality and the standardized mortality rate (SMR).

Although many concepts can be used to define nursing workload,^(^ ^16^ ^,^ ^17^ ^)^ this study employed the consensus definition of workload as the number of hours devoted by nursing professionals to the care of each patient. To calculate the nursing workload, the daily sum of scores for each patient was considered. In both the TISS-28 and the NEMS, the sum of the scores for each item reflect the nursing workload over a 24-hour period.^(^ ^18^ ^)^ The maximum scores for the TISS-28 and NEMS are 78 and 63, respectively.

**Figure 1:**
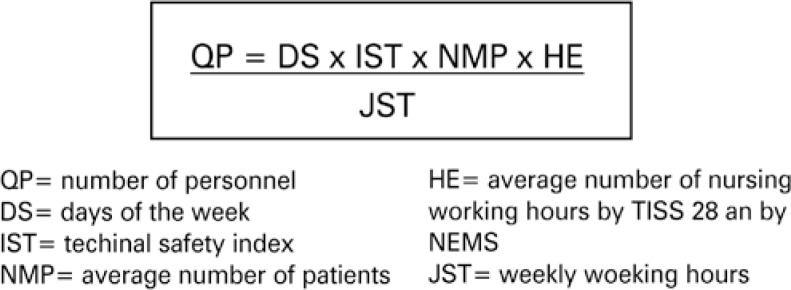
Formula for nursing manpower planning TISS-28: Therapeutic Intervention Scoring System-28; NEMS: Nine Equivalents of Nursing Manpower Use Score.

Each point on the TISS-28 and NEMS is equivalent to 10.6 minutes spent in direct care nursing activities during an 8-hour shift.^(^ ^1^ ^,^ ^2^ ^,^ ^7^ ^,^ ^18^ Thus, to estimate the nursing workload over 24 hours, the score was multiplied by 10.6^(^ ^18^ ^)^ and then by 3, and the result was divided by 60. To compare the effectiveness of each score for planning nursing staff levels, the guidelines of the Federal Council of Nursing (COFEN - *Conselho Federal de Enfermagem* ) were taken into consideration.^(^ ^19^ ^)^ These guidelines establish the legal parameters for the minimum number of nurses and nursing technicians required in each type of health care unit. The number of staff was calculated according to COFEN guidelines, in conjunction with TISS-28 and NEMS workload calculations. Figure 1 shows the formula used to determine the necessary number of staff according to the TISS-28 and NEMS workload estimations. Manpower planning was based on a 7-day, 36-hour workweek; a technical safety index of 15%; an average of 12 patients admitted to or hospitalized in the unit per day; and the average hours of nursing care estimated by TISS-28 and NEMS.

The study was conducted according to Declaration of Helsinki guidelines and was approved by the Ethics Committee of the organization where the study was conducted (protocol 06/03242). The informed consent requirement was waived for this study.

## RESULTS

The study included 459 children, yielding a total of 3,409 observations. The sample characteristics are presented in table 1 . Most patients were male, and 65% were under 5 years of age. The proportion of patients coming from internal and external referrals was similar. Most children were admitted through SUS. The expected mortality rate according to PIM-2 data was 6.6%, but the observed mortality rate was 7.2% (SMR=1.09; z<1.96).

**Table 1:** Characteristics of patients admitted to the pediatric intensive care unit

Variable		n (%)
Sex		
	Male	270 (59)
	Female	189 (41)
Age		
	28 days-1 year	155 (34)
	1-5 years	143 (31)
	5-12 years	123 (27)
	Above 12 years	38 (8)
Origin		
	Emergency room	179 (39)
	Operating room	147 (32)
	Ward	87 (19)
	Transfer from outside hospital	46 (10)
Payer		
	Brazilian Unified Health System	312 (68)
	Private health insurance	147 (32)
Mortality		
	Expected (PIM-2)	30.5 (6.6)
	Observed	33 (7.2)

PIM: Pediatric Index of Mortality.

**Figure 2:**
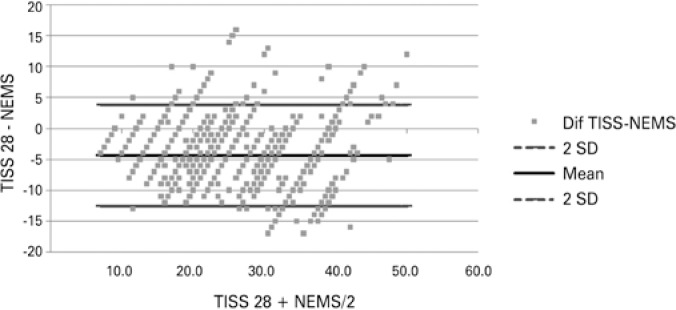
Agreement between mean TISS-28 and NEMS TISS-28: Therapeutic Intervention Scoring System-28; NEMS: Nine Equivalents of Nursing Manpower Use Score; SD: standard deviation.

The mean TISS-28 and NEMS scores obtained for the overall sample (3,409 observations) were 20.8±8.0 and 25.2±8.7 points, respectively. The mean difference between the TISS-28 and NEMS scores was −4.3±4.1. The limits of agreement for two standard deviations were +3.85 to −12.55. The difference among the scores greater than two standard deviations (>8.20) was only 5.7%, which demonstrated good agreement between the indicators ( Figure 2 ).

Based on the TISS-28 and NEMS scores obtained in this study, we calculated the mean nursing work hours per patient over a 24-hour period ( Table 2 ). The mean nursing workload estimates were 11 and 13.3 hours according to the TISS-28 and NEMS, respectively. The nursing staff required to meet the needs of the four pediatric ICU teams was estimated at 29.6 and 35.8 by the TISS-28 and NEMS averages, respectively.

**Table 2:** TISS-28 and NEMS corresponding estimated nursing workload and manpower requirements

	Score±SD	Working hours per shift±SD	Working hours in 24h±SD	Professionals required±SD
TISS-28, average	20.8±8.0	3.7±1.4	11.0±4.2	29.6±11.3
TISS-28, on admission	18.9±8.7	3.3±1.5	10.0±4.6	26.8±12.3
TISS-28, maximum	21.3±9.5	3.8±1.7	11.3±5.1	30.3±137
NEMS, average	25.2±8.7	4.4±1.5	13.3±4.6	35.8±12.3
NEMS, on admission	24.1±9.1	4.3±1.6	12.8±4.8	34.3±12.9
NEMS, maximum	26.4±9.8	4.7±1.7	14.0±5.2	37.5±14.0

TISS-28 and NEMS scores refer to the mean data from 3,409 observations. The TISS-28 and NEMS scores for admission and maximum refer to data from 459 patients on the first day of pediatric intensive care unit admission and on the day of highest score.

TISS-28: Therapeutic Intervention Scoring System-28; NEMS: Nine Equivalents of Nursing Manpower Use Score; SD: standard deviation.

**Figure 3:**
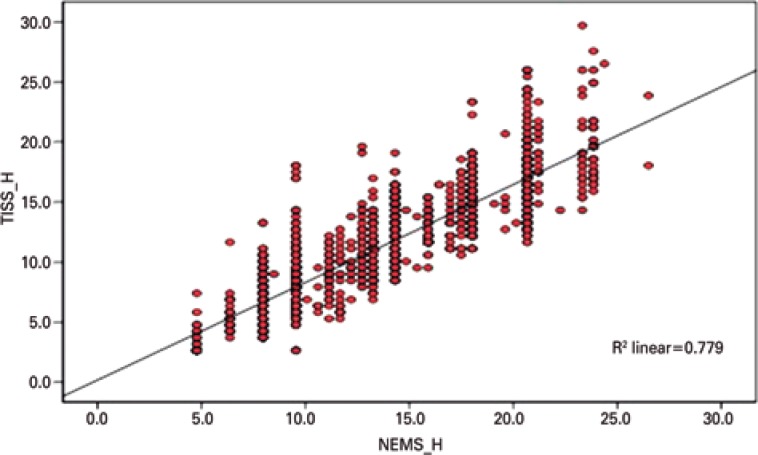
Correlation between TISS-28 and NEMS in hours of nursing work TISS-28: Therapeutic Intervention Scoring System-28; NEMS: Nine Equivalents of Nursing Manpower Use Score.

The TISS-28 and NEMS work hour estimates showed a positive and linear association, with a strong correlation coefficient (r) of 0.882 and determination coefficient (R^2^) of 0.779 for all 3.409 observations ( Figure 3 ). Furthermore, strong correlations were observed for the first day of pediatric ICU admission (r=0.891) and for the maximum score (r=0.904), p<0.01 for all observations.

## DISCUSSION

We estimated the nursing workload and required nursing staff levels in a pediatric ICU using the TISS-28 and NEMS scales. This comparison revealed that despite covering fewer items, the NEMS is at least as reliable as the TISS-28. Although we did not evaluate the time it took to collect data for the TISS-28 and NEMS scores, we believe that the NEMS, because it evaluates fewer items, requires less paperwork time, which would facilitate its daily systematic use in the pediatric ICU. A multicenter study evaluating the NEMS scale also concluded that it is highly accurate.^(^ ^20^ ^)^ This supports the use of the NEMS, a much simpler tool, in complex environments where recording the time required for each activity is itself cumbersome. Therefore, using a more compact scale might produce more reliable measurements, especially for a first-time assessment.

Both the TISS-28 and the NEMS cover items that are likely to be relevant for ICU all over the world, despite being developed for European ICU. The nine items on the NEMS scale were chosen based on the most representative nursing tasks from the same database used to validate the TISS-28.^(^ ^8^ ^)^

One limitation of scales such as the NEMS and even TISS-28 is that they do not take into account administrative tasks or activities related to family support or counseling, for example, which might be especially common in pediatric intensive care units. More recently, a promising scale developed to measure nursing workload is the Nursing Activities Score,^(^ ^21^ ^)^ and it includes these additional tasks. However, we believe that in units without data collection experience, the use of the NEMS might be easier until data collection becomes routine.

The mean NEMS score obtained in the present study was significantly higher than the mean TISS-28 score, which translated into a higher nursing workload. Another study performed in the same unit to validate the NEMS with pediatric patients found mean scores of 19.28 for the TISS-28 and 24.30 for the NEMS, similar to those reported in the present study.^(^ ^5^ ^)^ Moreover, a study performed in two adult ICUs, in the city of Porto Alegre (RS), found higher NEMS scores than TISS-28 scores both at admission and discharge.^(^ ^22^ ^)^

Conversely, in a study conducted with 55 adult patients admitted to a cardiac surgery recovery unit, over a 2-month data collection period, the average TISS-28 score was higher than the average NEMS score, as was the demand for nursing care.^(^ ^18^ ^)^ These results could be explained at least in part by the short duration of the study, the small sample size, and the fact that it only included surgical patients.

Several criteria can be used to define nursing workload: the nurse/patient ratio, job characteristics, the condition of hospitalized patients, and situations arising in the ICU. In the present study, we assessed “workload at the patient level,” *i.e* ., the condition of each patient based on the therapeutic interventions performed and measured by the TISS-28 and/or NEMS scores.^(^ ^17^ ^)^

Although the unit work shifts were 6 hours during the day and 12 hours at night (which would total up approximately 2.8 and 3.3 hours according to the TISS-28 and NEMS, respectively), we selected 8-hour shifts for our calculations to allow comparison between our results with those of other national and international studies. We found a mean of 3.7 and 4.4 hours of direct patient care per 8-hour shift, according to the TISS-28 and NEMS, respectively. Studies using the TISS-28 to focus on nursing workload in adult ICU found higher values.^(^ ^2^ ^,^ ^9^ ^)^ The time devoted to direct patient care ranged from 4.6 to 5.9 hours according to the length of hospital stay.^(^ ^2^ ^)^ In another study, when converting published values to TISS-28 scores, we found a mean of 4.1 hours per shift, ranging from 2.5 hours in the burn unit to 5.6 hours in the liver transplant unit.^(^ ^9^ ^)^

The pediatric ICU in this study had 46 nursing professionals available for direct patient care. According to the two scales, however, manpower planning should be lower: 29.6 and 35.8 according to the TISS-28 and NEMS, respectively. However, we realize that many authors consider that therapeutic intervention scores describe only half of the nursing workload in a 24-hour period,^(^ ^18^ ^,^ ^21^ ^)^ since these scores disregard tasks unrelated to direct patient care, as well as other physical, psychological, and organizational factors that add to the workload.^(^ ^23^ ^)^ In Brazil, ICU must comply with federal guidelines regarding minimum staffing as a function of the number of beds available.^(^ ^24^ ^)^ According to these guidelines, the present pediatric ICU would require at least 32 nursing professionals. However, this number does not take into account an additional 15% of staff needed to cover days off, vacations, and unplanned absences, as recommended by the COFEN.^(^ ^19^ ^)^ By adding the technical safety index of 15%, 36.8 personnel would be necessary.

The use of nursing workload measurement scales is useful for the management of human resources in pediatric ICU. It can also contribute to the distribution of personnel according to the needs of pediatric patients.

One limitation of this study is that it was conducted at a single pediatric ICU. Another limitation could be that the scales, which were developed for adults, may not reflect the practice of round-the-clock bedside parental presence and visitation in pediatric ICU settings or the time nurses spend supporting and communicating with parents, who may be present for much of the day. Furthermore, the TISS-28 and NEMS are subject to criticism, since they reflect therapeutic interventions performed on patients and do not cover important nursing activities, such as hygiene activities and managerial tasks. These items have been included in the Nursing Activities Score,^(^ ^21^ ^)^ which has already been used with promising results in one pediatric study.^(^ ^25^ ^)^ The paucity of studies with pediatric populations also limits comparison of the results. It is recommended that further studies regarding this topic and the issue of family-centered care practice be conducted at pediatric ICU to evaluate the effectiveness of these two nursing workload measurement scales.

## CONCLUSION

We found good correlation and excellent agreement between TISS-28 and NEMS scores in this pediatric patient population. The time that the NEMS estimated for nursing activities was significantly higher than that of the TISS-28. We believe that using the NEMS might be less cumbersome and produce reliable results, especially for first-time pediatric intensive care unit assessments.
